# From Online Learning to Clinical Practice: An Investigation on the Factors Influencing Training Transfer Among Physicians

**DOI:** 10.3390/healthcare13020133

**Published:** 2025-01-13

**Authors:** Giovanni Schettino, Vincenza Capone

**Affiliations:** Department of Humanities, University of Naples Federico II, 80133 Naples, Italy

**Keywords:** physicians, training transfer, theory of planned behaviour, training satisfaction, job autonomy

## Abstract

**Introduction:** Massive Open Online Courses (MOOCs) are an agile context for workplace training, which can provide physicians with needed knowledge and skills related to their clinical practice. From an organisational standpoint, their effectiveness can be assessed on physicians’ intention to transfer what they learn through them in the workplace. Despite the Theory of Planned Behaviour (TPB) standing among the more solid models in explaining individuals’ behavioural intention, its adoption in investigating the training transfer process among physicians is notably underdeveloped, limiting its contribution to enhancing the transfer rates of MOOCs content. **Method:** Based on such a consideration, the present study adopted an extended TPB model to investigate the potential psychosocial factors affecting the intention to transfer knowledge and skills learned through MOOCs in the workplace among 217 Italian physicians who completed an online self-report questionnaire. **Results:** Hierarchical regression analyses were performed, showing that among the TPB variables, perceived behavioural control and attitude significantly predicted transfer intention. The inclusion of training satisfaction and job autonomy in the model significantly increased the explained variance in intention. **Conclusions:** These findings have valuable implications for human resource development (HRD) in healthcare as they suggest the need to design MOOCs based on a flexible competency model in order to promote physicians’ engagement and commitment to adopting new knowledge and skills. Finally, interventions aimed at recognising physicians’ efforts in transferring learned content as well as empowering their ability to shape how they perform their professional duties could further enhance the impact of workplace training.

## 1. Introduction

Workplace training is a primary human resource development (HRD) strategy that consists of the employees’ systematic acquisition and development of knowledge and skills required to perform work-related tasks adequately [1,2]. It is pivotal to increasing organisational performance, enhancing work quality, encouraging teamwork, improving job satisfaction, and reducing errors [3,4,5,6]. More in detail, training has become an essential endeavour within organisations in times characterised by rapidly evolving job demands, roles, and work practices to achieve their goals [7], as it can support workers in developing resources to handle job demands effectively. This can mitigate the risk of stress and burnout [8], improving work engagement and, in turn, workers’ performance and well-being [9,10]. The strategy described benefits significantly from new technologies [11], notably Massive Open Online Courses (MOOCs), which have become a widely adopted online learning context, attracting over 220 million learners [12]. MOOCs are designed for a potentially unlimited number of students who can enrol in a course simultaneously with an Internet connection [13]. These courses are used mainly in higher education [14] and are typically developed by renowned education institutions [15], which define their content, such as short lectures and assignments, usually evaluated through automated systems [16]. This format substantially reduces costs relative to in-presence training programmes [17]. Furthermore, it provides trainees with great autonomy in learning and can offer an environment that encourages the development of ideas beyond the course content [18].

In the workplace, MOOCs are implemented in the form of corporate MOOCs. As Egloffstein and Ifenthaler [19] claim, these courses differ from their academic counterparts since they are limited to organisations and their employees and may include face-to-face elements as well as custom-built content. These features have made corporate MOOCs flexible, scalable, cost-effective, and high-quality training able to satisfy the professional development needs of healthcare workers (HCWs), including physicians [13,20,21,22]. However, it must be noted that corporate MOOCs require certain specific conditions to be effective [23]. Conversely, they may represent an investment that cannot deliver tangible benefits to organisations, particularly when the knowledge and skills they focus on do not translate into working behaviours or training transfer [23,24]. Therefore, training might fail to provide physicians with the knowledge and skills required to adequately face challenges related to a continuously evolving work context such as healthcare (e.g., rapid advancements in medical practice, evolving patients’ values about care, and shifts in policies), consequently hindering the ability of organisations to deliver high-quality care [25,26,27]. Despite the relevance of this issue, the literature on training transfer in relation to corporate MOOCs aimed at physicians is a relatively underdeveloped area of research [13], limiting in such a way the proper identification of factors promoting the effectiveness of these training programmes.

In light of the above, a primary goal of our research consisted of bridging this literature gap by identifying which factors can influence the effectiveness of MOOCs in shaping the training transfer process among physicians. Specifically, this study adopted the Theory of Planned Behaviour (TPB) [28] due to its proven ability to explain the decision-making process underlying behavioural intention [29]. In doing so, we aim to provide useful evidence to enhance the outcomes of training-related organisational interventions. More specifically, the present study investigated the role of attitude towards training transfer, subjective norms related to supervisors and colleagues, and perceived behavioural control in forming physicians’ intention to implement the transfer behaviour. Additionally, consistent with the organisational literature on training transfer that has suggested the complex nature of this process [30,31], we assessed whether training satisfaction and job autonomy contributed to explaining the decision-making process under investigation.

### 1.1. Theoretical Framework

Training transfer, as postulated by Baldwin and Ford [23], refers to the “degree to which trainees effectively apply the knowledge, skills, and attitudes gained in a training context to the job” (p. 63). In formal training settings [32], including corporate MOOCs, training transfer reflects the adoption of skills and knowledge acquired during such training courses in the workplace.

Attention must be drawn to the consideration that training transfer is a critical aspect of knowledge management, which reflects the process of creating, sharing, and using knowledge within an organisation [33]. Studies have shown that organisations implementing an effective knowledge management system (KMS) can improve both knowledge sharing (a set of behaviours mainly related to informal learning involving the exchange of experiences, skills, and knowledge across organisational members) [34,35,36,37] and training transfer [38]. In this vein, organisational structure and its processes are crucial in promoting an effective transfer of learning [39]. However, since that training transfer is a complex process, it is sensitive not only to work-related factors but also to trainees’ characteristics and training design, as highlighted by Baldwin and Ford [23] and subsequent research [24,31,40,41].

Given this complexity, the TPB can be regarded as a suitable theoretical framework for elucidating the multifactorial nature of training transfer. Specifically,, the TPB has been widely recognised as one of the most effective theories in explaining the decision-making process underlying individuals’ behaviours [29]. Furthermore, it has demonstrated its usefulness in understanding the different facets of the training transfer process [42,43,44,45]. A main factor of the TPB is intention, which is considered the most proximal antecedent of behaviour. More precisely, intention consists of the individuals’ decision to exert effort to perform a certain behaviour [46]. Transfer intention, consequently, reflects the degree to which individuals are willing to adopt newly acquired knowledge and skills [47,48], and it is strongly associated with the transfer behaviour. In support of such a thesis, a study by Quesada-Pallarès et al. [49] investigated the adoption of the content of online courses among a sample of Spanish employees and documented that transfer intention was positively associated with the transfer behaviour.

According to the TPB [28], intention is, in turn, determined by the following three factors: attitude; subjective norms; and perceived behavioural control (PBC).

Attitude can be defined as an overall positive or negative evaluation of performing a given behaviour. Thus, attitude towards the transfer of training can be conceived as the trainees’ more or less favourable assessment of integrating training into their profession [50]. It should be emphasised that attitude is a manifestation of expectations about job performance and judgements about the value of outcomes associated with the adoption of training in work practice [28,51]. In this regard, previous studies have demonstrated a positive association between the anticipation of attaining desired outcomes through the transfer of training into work and attitude towards transfer [51,52]. The Learning Transfer System Inventory [24] corroborates this view, suggesting that workers are more likely to form a favourable transfer intention when they expect the inherent behaviour to enhance their job performance.

Subjective norms are defined as the personal perceptions of pressures or social expectations related to the adoption of a given behaviour. Prior studies have reported that subjective norms can predict different behavioural intentions regarding work, including transfer intention [51,53,54]. More precisely, training outcomes can be shaped by the perceived support of the supervisors and colleagues as well as their pressure on training-related behaviours [31,49,55,56,57]. For example, Burke and Hutchins [58] highlighted the dual role of trainers, peers, and supervisors as facilitators or barriers to learning transfer. Moreover, as Cheng [43] claims, the relationships of trainees with these people can be considered a measure of the transfer climate. Therefore, when individuals perceive that significant others in their job expect them to perform the transfer behaviour, they tend to report an increased willingness to apply knowledge and skills mastered to their work.

Finally, PBC is the perception of the extent to which performing a behaviour is within one’s control or, in other words, the belief about how easy or difficult performing a behaviour, such as the training transfer, is [45]. Specifically, Ajzen [28] argued that self-efficacy and controllability jointly determine PBC. In this vein, such a TPB component is a manifestation of internal and external factors, including those related to working practices [59], shaping the transfer intention.

Although the proven reliability of the TPB in explaining a number of behaviours [29], its adoption in investigating HCWs’ implementation of new knowledge and skills following participation in corporate MOOCs has been extremely limited. More precisely, Singh et al. [47] adopted a classic TPB model in a sample of Indian medical teachers, showing that the model explained 41% of the variance of intention. Participants’ intention to transfer learning was affected mainly by attitude (β = 0.51) and PBC (β = 0.21), while subjective norms showed a negligible effect (β = 0.07). Similarly, Faraji Dehsorkhi et al. [60] conducted an empirical study involving Iranian nurses and aimed to test a classic TPB model. The authors documented that attitude toward training transfer was the main antecedent of transfer intention (β = 0.52), followed by PBC (β = 0.38) and subjective norms (β = 0.25). Moreover, intention was the primary predictor of transfer behaviour (β = 0.55). This model accounted for 62% of the variance in transfer intention and 52% in transfer behaviour. A subsequent study [61] with a qualitative methodology adopted the TPB to examine Irish physicians’ attitudes and beliefs towards programmes for maintaining professional competence. The findings highlighted that physicians who perceived the positive impact of such training on their job reported higher intention to attend the training programmes and adopt knowledge and skills learned.

It is noteworthy that the above-mentioned studies adopted a classic TPB model. This strategy leads to at least three considerations. First, as Conner and Norman [62] argued, it may limit the predictiveness of the TPB compared to an approach that includes additional variables. Second, focusing solely on the TPB core constructs does not allow for a proper evaluation of further factors—such as those related to training and job design characteristics—that shape physicians’ working experience within healthcare organisations and, in turn, their professional behaviours. Moreover, as additional factors can provide a more comprehensive explanation of behaviour, this can result in identifying more clearly which factors should be leveraged to improve outcomes of interventions, including those related to training aimed at physicians.

Concerning these factors, prior studies have regarded training design as one of the crucial antecedents of transfer behaviour [41,51,63,64,65]. It consists of the degree to which training has been designed and delivered in a way that allows trainees to transfer learning to the job [24]. Training design is positively associated with satisfaction with training, which can be conceptualised as employees’ appreciation for this activity. In turn, such a perception can promote employees’ learning retention and their readiness to transfer learning [63,66]. With this regard, Tzafilkou et al. [67] demonstrated that teachers’ satisfaction with information and communications technology training affected their intention to transfer learning to their jobs. It must be noted that the benefits of this kind of satisfaction are not limited to the context of training, as demonstrated by several studies. For instance, Schmidt [68] showed that training-related factors (e.g., training methodologies, training content) are essential in enhancing employees’ satisfaction with training programmes and, in turn, such a perception can predict overall job satisfaction, an indicator of workplace well-being [69], which Spector [70] regards as the extent to which people like or dislike their jobs. A subsequent study [63] supported the broad impact of training satisfaction, reporting that training satisfaction was associated with higher organisational commitment and, in turn, greater readiness to transfer workplace training content, as well as reduced absenteeism rates.

In addition to training satisfaction, a large body of research has identified a number of factors related to the design of the working activity [71,72,73,74]. Such factors include job autonomy, which refers to the degree of discretion individuals have in making decisions about how to carry out their work tasks [75]. As Axtell et al. [72] suggested, job autonomy is a key aspect of effective training transfer as it helps overcome barriers related to learning adoption. Individuals with more job autonomy can access information, support, resources, and growth opportunities that can boost their level of control [76]. This condition can, in turn, facilitate the challenges associated with integrating new learning into their work practices. In support of this argumentation, Awoniyi et al. [74] proved that job autonomy predicted the intention among workers employed in various sectors—including community-based organisations, community development corporations, financial institutions, and government agencies—to transfer the content of a training programme.

Despite the highlighted essential role of training satisfaction and job autonomy in shaping training outcomes, with regard to physicians, as far as we know, no study has included training satisfaction and job autonomy in a model to explain transfer intention. In addition, we did not find evidence about the consideration of these factors in an extended TPB model regarding such an intention.

### 1.2. Aim and Hypotheses

**Figure 1:**
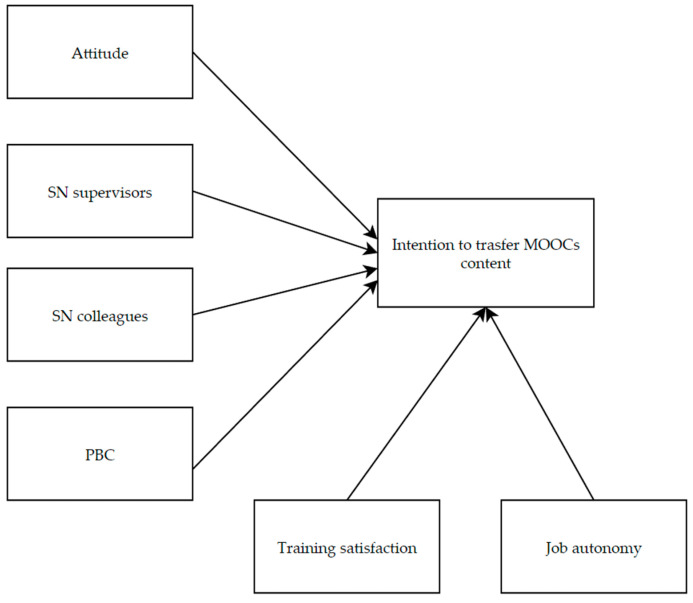
Hypothesised relationships among the psychological variables.

Since MOOCs have become a widely adopted tool in the context of workplace training of physicians, it is essential to identify the factors contributing to their success. Accordingly, the current study aimed to provide a better understanding of individual-, training-, and organisational-level key factors [24,30] that could affect the physicians’ intentions to transfer knowledge and skills they learn through these online courses to clinical practice as well as the underlying decision-making process. Following this reasoning, we adopted an extended TPB model (Figure 1) based on the proven effectiveness of such a theoretical framework in explaining the process leading to forming a behavioural intention [47,51,62]. We hypothesised that transfer intention would have been positively predicted by attitude (H1), subjective norms related to supervisors (H2a) and colleagues (H2b), and PBC (H3).

Furthermore, we considered two additional predictors of intention—namely, training satisfaction and job autonomy—to improve the explanatory power of the hypothesised model and, thereby, better support HRD interventions in optimising outcomes of healthcare organisations’ training-oriented efforts. Therefore, consistent with the literature on training transfer [63,74], we hypothesised that training satisfaction and job autonomy would have been direct predictors of physicians’ intention to adopt MOOCs content in the workplace (H4).

## 2. Materials and Methods

### 2.1. Procedure and Participants

The current cross-sectional study employed an online self-report questionnaire, which took about 15 min to complete. The questionnaire was created through the Qualtrics platform since it allows for high flexibility and provides tools for preliminary identification of low-quality responses (e.g., multiple submissions from the same individuals).

In order to take part in this study, participants were required to be (1) of legal age (age ≥ 18), (2) Italian physicians employed in public or private healthcare organisations, and (3) have attended a corporate massive open online course during the prior two years. The questionnaire link was shared through informal channels (e.g., social network groups). The administrators of the selected groups and pages were informed about the nature and objectives of this study and gave their approval to share the questionnaire link.

Before recruiting participants, a power analysis was conducted using G*Power [77] to determine the sample size needed to detect a small effect size (*f*^2^ = 0.15) in a hierarchical regression analysis, including 6 psychological and 13 socio-demographic predictors. This analysis was based on an alpha level of 0.05 and a power of 0.80, yielding an estimated sample size of *N* = 153. A total of 217 physicians met the inclusion criteria and completed the questionnaire after being informed about the anonymity of data collection and giving informed consent. Therefore, the sample size appears more than adequate for testing the statistical hypotheses. Participation was voluntary, and no incentive for participation was offered. Data were collected from December 2022 to February 2023. This study was conducted following the receipt of ethical approval by the Ethics Committee of Psychological Research of the University of Naples Federico II. These data are part of a larger dataset used for a study published previously to investigate different hypotheses [59].

Participants were almost equally distributed by gender (men = 59%), aged between 26 and 70 years (*M* = 49.3; *SD* = 11.6). Of these, 53.9% came from South Italy and the Islands, 26.3% from Northern Italy, and 19.8% from Central Italy. Most of them had managerial responsibilities (65.9%) and were employed in public healthcare organisations (77.9%) for an average of 11.1 years (*SD* = 9.5). Moreover, 87.8% had a master’s degree, and 12.2% had a Ph.D. degree. Regarding medical specialisation, 28.5% were general practitioners; 12.5% were oncologists; 8.2% were internists; 6.3% were paediatricians, and 44.5% were practised in other specialities. Respondents had completed an average of 8.4 (*SD* = 7.2) MOOCs aimed at enhancing hard (91.7%) or soft (8.3%) skills related to their jobs. These courses had an average length of 8.6 h (*SD* = 8.1) and were mainly held by male instructors (96.3%). Finally, 44.2% of physicians attended MOOCs by using their desktop computers. 

### 2.2. Measures

The initial section of the questionnaire required participants to give informed consent. Thereafter, they were instructed to answer all subsequent questions by considering corporate MOOCs related to their medical profession. Measures included in the questionnaire were administered to all participants in the same order. All the answers to the questionnaire were mandatory, so there were no missing values. TPB items were developed based on the guidelines formulated by Ajzen [78]. More in detail, following the principle of compatibility [79], predictors (attitude, subjective norms, and PBC) and intention were measured at the same specificity level to maximise the predictive power of the TPB.

Physicians’ intention to transfer MOOCs content in their workplace was assessed with three items [78] evaluated on a 5-point Likert scale from “completely disagree” (1) to “completely agree” (5): “I intend to use knowledge or skills learned through MOOCs on the job”; “I plan to use knowledge or skills learned through MOOCs on the job”; “I will use knowledge or skills learned through MOOCs on the job”. Cronbach’s α for the current study = 0.94.

Physicians’ attitude towards the transfer of MOOCs content was assessed with five items [78] evaluated on a semantic differential scale ranging from 1 (negative pole) to 5 (positive pole). Specifically, “Applying the knowledge or skills learned through MOOCs on the job is disadvantageous–advantageous; negative–positive; not important–important; unsatisfactory–satisfying; not valuable–valuable”). Cronbach’s α for the current study = 0.94.

Subjective norms were assessed with four items [78] evaluated on a 5-point Likert scale from “completely disagree” (1) to “completely agree” (5). Two items were adopted to evaluate the perceived pressures participants perceived from supervisors (SN supervisors), and two items from colleagues (SN colleagues): “My supervisors think that I should use knowledge or skills learned through MOOCs on the job”; “My supervisors expect me to use knowledge or skills learned through MOOCs on the job”; “My colleagues think that I should use knowledge or skills learned through MOOCs on the job”; “My colleagues expect me to use knowledge or skills learned through MOOCs on the job”. Cronbach’s alphas for the current study = 0.91 (SN supervisors) and 0.92 (SN colleagues).

Perceived behavioural control about training transfer was assessed with 4 items [78] using a Likert scale ranging from 1 = “completely disagree” to 5 = “completely agree” (i.e., “For me, it is possible to use knowledge or skills learned through MOOCs on the job”; “It would be easy for me to use knowledge or skills learned through MOOCs on the job”; “Using knowledge or skills learned through MOOCs on the job depends solely on me”; “Using knowledge or skills learned through MOOCs on the job is within my control”). Cronbach’s α for the current study = 0.89.

Training satisfaction was assessed using 1 item from the Course Experience Questionnaire (CEQ) [80]. The instrument evaluates the perceived satisfaction of training through a 5-point Likert scale ranging from 1 = “completely disagree” to 5 = “completely agree”. The item was adapted to be suitable for the MOOCs context (i.e., “Overall, I am satisfied with the quality of MOOCs”).

Job autonomy was assessed using the 6-item Job autonomy subscale of the Majer-D’Amato Organisational Questionnaire 10 (M-DOQ10) [81]. The instrument evaluates the perceived job autonomy individuals experience during their work tasks through a 5-point scale ranging from 1 = “false” to 5 = “true” (e.g., “In my work, I have a certain degree of autonomy”). Cronbach’s α for the current study = 0.94.

Finally, we collected information about participants’ socio-demographic characteristics, including age, gender, education, medical speciality, job context, job role, organisational tenure, and the Italian region of residence. We also asked questions about their experiences with MOOCs: the number of corporate MOOCs completed during the two previous years; the specific contents of such courses (MOOCs focused on hard or soft skills); their length; the gender of instructors; and the device they used to attend these courses.

### 2.3. Data Analysis

Statistical analyses were conducted using SPSS 29 and R (https://www.r-project.org/ (accessed on 4 April 2023)). A preliminary step to test our hypotheses consisted of carrying out a confirmatory factor analysis (CFA) and verifying the convergent and discriminant validity of the constructs. Notably, convergent validity was evaluated by examining the Average Variance Extracted (AVE) of the constructs, which should be equal to or greater than 0.5 [82]. Discriminant validity was assessed using the Fornell–Larcker criterion, comparing the square root of the AVE with the correlations between latent constructs. Additionally, correlations were inspected to evaluate potential common-method bias [83].

Descriptive analyses were performed on all study variables. Moreover, correlations were carried out to evaluate the associations between psychological variables.

In order to identify potential predictors of behavioural intention, a hierarchical regression analysis was conducted. The analysis focused solely on transfer intention as the dependent variable and included three blocks of predictor variables. Based on Barbaranelli’s [84] recommendations, we first ascertained the following assumptions required for regression analysis: (1) linearity of the relationships between the independent variables and dependent variable; (2) absence of multicollinearity among the independent variables; and (3) normality distribution of errors. The first assumption was confirmed through a visual inspection of scatterplots by plotting the dependent variable against each independent variable. The second assumption was evaluated by analysing collinearity statistics, which reported that predictors had variance inflation factor (VIF) coefficients below 3.66, suggesting a minimal presence of multicollinearity [85]. Lastly, the normality of the distribution of residuals was evaluated by analysing the alignment of residual distribution graphs (histogram and P-P plot) with a normal distribution.

As these assumptions were verified, we added the independent variables in three blocks, adopting an approach consistent with previous studies [86,87]. The first one consisted of socio-demographic variables. The second block added TPB constructs: attitude; SN supervisors; SN colleagues; and PBC. Finally, the third block added training satisfaction and job autonomy. A significant F change (*p* < 0.05) indicated that adding variables significantly improved the model prediction.

## 3. Results

### 3.1. Reliability and Validity of the Measures

The results of the measurement model (Table A1) showed strong relationships between the latent constructs and items with factor loadings > 0.5 [88], notably ranging from 0.71 to 0.96, hence suggesting the validity and reliability of the constructs. Moreover, the convergent validity was supported since the AVE for all constructs was above the minimum threshold values. Likewise, the results of the Fornell–Larcker criterion (Table A2) indicated that the discriminant validity of the constructs was established. Additionally, we examined the potential presence of common method bias in the data and found no correlation greater than the 0.90 threshold [83].

### 3.2. Descriptive Analyses

Descriptive statistics and correlations between the psychological variables are shown in Table 1. Participants reported above-average levels in all TPB constructs, with behavioural attitude scoring the highest. Furthermore, both training satisfaction and job autonomy showed moderate levels. Finally, positive and significant correlations emerged between all psychological variables (*p* < 0.01).

### 3.3. Hierarchical Regression Analysis

The results of the hierarchical regression analysis are displayed in Table 2. Model 1, which only included socio-demographic variables, explained 22% of the variance in intention. Specifically, participants’ age, gender, education, job context, and the number of MOOCs completed influenced their intentions to adopt the learning acquired. The introduction of TPB variables in Model 2 yielded a substantial increase in the explained variance, accounting for 73%. Among the TPB constructs, PBC, followed by attitude, emerged as significant and positive predictors of intention, while subjective norms were not significantly related to intention. Finally, regarding Model 3, training satisfaction and job autonomy positively and significantly influenced intention. More in detail, these variables led to a further significant increase in explained variance, with R^2^ = 76%. Consequently, Model 3 showed the best fit. It is important to note that in this final model, attitude and PBC were still significant predictors of intention. As for socio-demographic variables, age solely impacted transfer intention in this model.

## 4. Discussion

The widespread popularity of MOOCs in workplace learning [13] and the pivotal role of transferring knowledge for achieving healthcare organisational goals [4,22] led us to investigate the inherent transfer process among physicians. Specifically, this current study aimed at providing a better understanding of the psychosocial factors affecting the intention of Italian physicians to apply corporate MOOCs content into clinical practice. In line with the seminal contribution by Baldwin and Ford [23], who conducted a number of inspiring studies on training transfer, and the evidence from the literature [31,40,41], we considered three categories of antecedents of transfer intention: individual-related variables; training characteristics; and work environment factors. These variables were included in an extended TPB model since it is well-acknowledged that such a theoretical framework can explain the process leading to behavioural intention formation [62].

Regarding the antecedents of intention, the core TPB components emerged as able to explain a substantial portion of the variance in physicians’ intention to apply MOOCs content in their workplaces. More precisely, analyses reported that attitude and PBC were significant predictors of intention, confirming H1 and H3. Additionally, results identified PBC as the stronger predictor of transfer intention. However, in contrast with H2a and H2b, subjective norms did not significantly predict intention.

As for H1 and H3, the findings are consistent with the literature identifying attitude towards transfer and PBC as two key antecedents of transfer intention [42,43,44]. Specifically, due to the fact that attitude includes both the evaluation of specific behaviour and its consequences, it is reasonable to suppose that physicians who expect a positive impact of adopting learning on their jobs are more likely to be willing to perform such a behaviour. Despite a positive attitude being essential in the transfer process, the findings suggest that having a high perceived control over transfer behaviour is a factor stronger associated with intention. Looking at Ajzen’s [89] conceptualisation of PBC, this perception can be determined by both internal and external barriers or constraints to a certain behaviour. Applying this line of reasoning to the interpretation of our findings, it is likely that physicians considered transferring MOOCs content in their clinical practice as potentially challenging, while at the same time, they perceived having the necessary control as emerged by the moderate levels of PBC they reported (Table 1).

In relation to subjective norms, the lack of a statistically significant relationship between them and transfer intention has been frequently observed in empirical research on training transfer [43,44,45,51], suggesting that such a TPB construct tends to report the lower predictiveness power of behavioural among other TPB core factors. Additionally, it must be underlined that our study focused on subjective norms related to supervisors and colleagues. This approach could have constrained the consideration of different nuances of such a TPB component. Specifically, potential sources of influence might also be non-medical organisational members or the broader social community.

In line with H4, the inclusion of training satisfaction and job autonomy within the model significantly increased the explained variance in physicians’ transfer intention. 

As for the former variable, our study suggests the potential to extend the conclusions of prior studies [64,65,67] to the physician population. Moreover, this result supports the thesis of various training transfer models [90,91], outlining the need to focus on training programme development to enhance its benefits at the organisational level. Indeed, the literature has consistently proven that a positive reaction to training could result in increased learning retention [63,66] and training transfer [63]. In this vein, our findings lead us to suppose that when physicians view training as designed in a functional way to be adopted in improving their job performance, they may be more committed to learning MOOCs content and, in turn, they may be more likely to form a stronger intention to integrate learned knowledge and skills into their professional behaviour. This speculation is supported by a growing body of studies that have come to the same conclusion, although conducted among different professional populations [92,93,94]. Therefore, it can be assumed that training content should be aligned with trainees’ specific professional needs and goals to maximise the likelihood of its adoption. 

As for the latter variable, the findings support the thesis about the crucial role of the work environment in enabling training transfer. More precisely, how healthcare organisations define the structure of work processes seems crucial in providing physicians with the resources required to implement new knowledge and skills in their jobs. Hence, it can be argued that physicians perceiving a higher level of autonomy in deciding how to accomplish work tasks can engage with learning MOOCs content and the consequent integration in their professional role according to their preferences. In other words, this autonomy ensures physicians can choose the course that better fits their learning and professional needs and, later, have the freedom to find opportunities to implement the learned skills [72], resulting in a higher transfer intention.

Lastly, an additional result concerns the role of age in predicting intention. More precisely, a younger age emerged as a significant factor affecting physicians’ decision to use what they had learned through MOOCs. This result is not particularly surprising since it is in line with evidence from previous studies on training transfer [95,96] and can be explained by considering different kinds of factors. First, it is widely recognised that effective online training requires adequate digital literacy [97,98,99], which tends to be lower among elderly people [100]. Additionally, older individuals experience a progressive decline in cognitive function, including working memory [101,102,103], which can negatively impact the self-regulation process [104], characterising learning in the context of MOOCs [105]. Taken together, these phenomena can explain the negative association between age and transfer intention of MOOCs content that emerged in the current study. Moreover, as indicated in a meta-analysis by Colquitt et al. [106], this negative relationship may be caused by both managers’ and self-perceptions. On the one hand, managers may have ageing-related prejudices or ageism [107] since they tend to perceive that the employees’ ability and training motivation diminish with advancing age. On the other hand, the fear of failure tends to increase with age, preventing older individuals from seeking training opportunities.

It is worth noting that the findings described must be interpreted in light of the limitations of this study. One such limitation is relying on convenience sampling, which limits the generalisability of the results to the broader population of physicians. Furthermore, the correlational design employed in this study does not allow for inferring cause-and-effect relationships. For this reason, further studies are required to confirm the directionality of the investigated relationships. In addition, this study focused on intention, not including a behavioural measure. Adopting a longitudinal design could clarify whether, over time, transfer intention determines an actual behaviour, following the indications from Holton III and Baldwin [108] when evaluating training transfer. Furthermore, since the hypotheses about subjective norms were not confirmed, future studies could explore the roles of different facets of such a TPB component (i.e., descriptive and injunctive norms), as well as further organisational variables not included in the current research (e.g., job satisfaction). Finally, while acknowledging the extensive evidence highlighting the essential role of informal learning in healthcare settings [36,39,109], the current study evaluated the effectiveness of training based on training transfer process. In view of the above, future research could expand upon these findings by including knowledge sharing as an additional measure to assess the effectiveness of corporate MOOCs in promoting physicians’ continuous professional development.

## 5. Conclusions

The present study provides a valuable contribution to the existing body of knowledge on training transfer in healthcare by employing a robust theoretical framework (i.e., the TPB) to understand the factors affecting physicians’ decisions to implement corporate MOOCs content in their organisations. Specifically, the findings suggest that PBC and attitude towards training transfer are crucial in forming physicians’ intentions to use new skills and knowledge learned through corporate MOOCs. Moreover, integrating additional variables to the core TPB constructs underlined that physicians’ satisfaction with training and the degree of perceived autonomy in their jobs could affect the transfer intention.

At a theoretical level, our results support the utility of TPB in explaining training transfer intention among physicians. They also highlight the need to extend the model beyond its core constructs to capture the different nuances of the training transfer process within healthcare organisations. In this respect, as far as we know, no prior studies have evaluated physicians’ satisfaction with corporate MOOCs and job autonomy as potential antecedents of transfer intention by adopting the TPB.

As for the practical implications, this study suggests that HRD practices within healthcare organisations should be inspired by an integrated approach to enhance the likelihood that training programmes can contribute effectively to organisational goals. This standpoint means considering the three main aspects of training transfer (i.e., individual variables, training characteristics, and work environment factors) [90,110,111]. More specifically, promoting positive attitudes related to the training application of MOOCs content appears to be advisable to encourage physicians to use what they have learned once the course programme ends. In this vein, training programmes should be designed based on a dual focus: taking into account the skills and knowledge already acquired by target participants and the specific training needs related to their professional roles. This approach could ensure that MOOCs content is both relevant and applicable, thereby promoting trainees’ engagement and enhancing the likelihood that what they learn is transferred to the workplace. Moreover, in order to be effective, such a strategy should be supported by a broader intervention aimed at redesigning competency models within healthcare organisations. In particular, given the continuous evolution of the healthcare field, competency models should be systematically reevaluated and updated [112] to be suitable in supporting physicians to cope with job-related demands placed on them [113]. Based on our findings, healthcare organisations could also optimise training effectiveness by rewarding the efforts of physicians engaging in the transfer behaviour as well as granting them greater autonomy in the different facets of their jobs, including training. This involves giving them more decision-making authority, flexibility in their work schedules, and higher opportunities and flexibility to participate in continuing professional development activities. Overall, our results reinforce the importance of the co-construction of organisational processes in healthcare to enhance physicians’ control over their jobs and, in turn, training outcomes [114]. The learning autonomy offered by MOOCs, along with the above-mentioned organisational resources, could be a way to promote both learning and adoption of new knowledge and practices to continuously improve physicians’ performances and the value of services provided to users [115]. Lastly, interventions addressing ageism [116] in healthcare organisations could encourage a more inclusive approach to training across age groups.

In conclusion, the described strategy might have the potential to improve training effectiveness, increase the return on investment in HRD within healthcare organisations, and, above all, improve physicians’ well-being, performance, and quality of care.

## Figures and Tables

**Table 1:** Descriptive analyses and correlations among the psychological variables.

	M (SD)	1.	2.	3.	4.	5.	6.
Intention	3.41 (0.92)	1					
2. Attitude	3.79 (1.12)	0.77 **	1				
3. SN supervisors	3.15 (1.02)	0.56 **	0.54 **	1			
4. SN colleagues	3.01 (1.04)	0.60 **	0.59 **	0.77 **	1		
5. PBC	3.33 (1.00)	0.77 **	0.67 **	0.49 **	0.57 **	1	
6. Training satisfaction	3.20 (0.90)	0.67 **	0.71 **	0.41 **	0.43 **	0.57 **	1
7. Job autonomy	3.82 (0.94)	0.50 **	0.51 **	0.36 **	0.42 **	0.64 **	0.37 **

Note. ** *p* < 0.01; M = mean; SD = standard deviation.

**Table 2:** Hierarchical regression analysis on behavioural intention.

Independent Variables	Model 1 β	Model 2 β	Model 3 β
Step 1: Demographic variables			
Age	−0.20 *	−0.15 **	−0.14 **
Gender	−0.19 **	−0.06	−0.04
Italian region of residence	−0.08	0.01	−0.01
Education	−0.23 ***	−0.10 *	−0.08
Medical specialty	0.02	0.01	0.01
Job context	−0.26 ***	−0.04	−0.07
Organisational tenure	0.06	0.01	0.01
Job role	0.13	0.03	0.06
MOOCs completed	0.26 ***	−0.02	−0.04
MOOCs length	0.05	0.05	0.01
MOOCs topic	0.03	0.07	0.01
Instructor’s gender	−0.03	−0.03	0.04
Device	−0.14	−0.02 ***	−0.04
Step 2: TPB variables			0.01
Attitude toward transfer	-	0.40 ***	0.20 **
SN supervisors	-	0.06	0.06
SN colleagues	-	0.07	0.07
PBC	-	0.42 ***	0.37 ***
Step 3: Additional variables			
Training satisfaction	-	-	0.12 *
Job autonomy	-	-	0.19 *
F-statistics	5.497(_13,189_) ***	33.833(_17,185_) ***	33.836(_19,202_) ***
Adjusted R^2^	0.22	0.73	0.76
Δ*R*^2^	-	0.48	0.02
Δ*F*	-	91.65 ***	9.00 ***

Note. *** *p* < 0.001; ** *p* < 0.01; * *p* < 0.05. Categorical demographic variables were dummy-coded as follows: gender: 1 = women, 0 = other; Italian region of residence: South of Italy = 1, other residence = 0; education: master’s degree or higher qualification = 1, other = 0; medical specialty: yes = 1, no (general medicine) = 0; job context: public health organisations = 1, other = 0; job role: managerial responsibility = 1; no managerial responsibility = 0; MOOCs topic: hard skills = 1, soft skills = 0; device: 1 = computer; 2 = mobile device.

## Data Availability

The dataset that supports the findings of this study is available from the corresponding author upon reasonable request.

## Appendix A

**Table A1:** Standardised factor loadings.

Variable	Λ					
	INT	ATT	SN SUP	SN COLL	PBC	JOB AU
INT.1	0.89					
INT.2	0.92					
INT.3	0.96					
ATT.1		0.78				
ATT.2		0.91				
ATT.3		0.95				
ATT.4		0.91				
ATT.5		0.83				
SN SUP.1			0.89			
SN SUP.2			0.94			
SN COLL.1				0.94		
SN COLL.2				0.93		
PBC.1					0.85	
PBC.2					0.85	
PBC.3					0.75	
PBC.4					0.81	
JOB AU.1						0.91
JOB AU.2						0.85
JOB AU.3						0.88
JOB AU.4						0.88
JOB AU.5						0.91
JOB AU.6						0.71

Note. INT = Intention; ATT = Attitude; SN SUP = Subjective norms (supervisors); SN COLL = Subjective norms (colleagues); PBC = Perceived behavioural control; JOB AU = Job autonomy.

**Table A2:** Discriminant validity with the Fornell–Larcker criterion.

	1.	2.	3.	4.	5.	6.
1. Intention	**0.92**					
2. Attitude	0.80	**0.89**				
3. SN supervisors	0.61	0.59	**0.92**			
4. SN colleagues	0.64	0.63	0.86	**0.93**		
5. PBC	0.87	0.77	0.63	0.66	**0.81**	
6. Job autonomy	0.80	0.84	0.56	0.61	0.79	**0.86**
*AVE*	0.85	0.79	0.85	0.87	0.66	0.74

Note. Diagonal elements highlighted in bold represent the square root of AVE.
